# Chlorhexidine mouthwash and augmentin to prevent Alveolar Osteitis after removal of mandibular third molar: a three-arm placebo- controlled randomized clinical trial

**DOI:** 10.4314/ahs.v24i2.14

**Published:** 2024-06

**Authors:** Salah Sakka, Mohamed Yasser Kharma, Ali Al Rafedah

**Affiliations:** 1 Department of Oral and Maxillofacial Surgery and Diagnostic Sciences, Prince Sattam Bin Abdulaziz University, Alkharj, KSA; 2 Specialist Oral and Maxillofacial Surgery, Jeddah, KSA

**Keywords:** Chlorhexidine, Augmentin, Alveolar osteitis, third molar, extraction

## Abstract

**Background:**

Alveolar Osteitis (AO) is a complicated problem that particularly occurs following the surgical removal of teeth resulting in severe pain and repeated practice/hospital visits.

**Objectives:**

The aim of the study was to investigate the role of postoperative Chlorhexidine (CHX) plus Augmentin in preventing Alveolar Osteitis AO after mandibular third molars removal.

**Methods:**

A total of 191 patients were randomly allocated to the CHX group (66 patients) or CHX and Augmentin group (63 patients) and Placebo group (62 patients). One mandibular third molar was removed in one session where surgical standard procedure was followed. All patients were prescribed rescue medication for postoperative pain relief. A postoperative follow up examination was performed on the third and seventh day to evaluate the existing cases of AO.

**Results:**

Group 2 (CHX and Augmentin) showed a significant reduction in AO (P < 0.05) when compared with group 1 (CHX) and group 3 (placebo). Patients have reported CHX side effects like taste alteration, bad taste, and staining.

**Conclusion:**

The combination of CHX plus Augmentin may be useful in reducing the incidence of AO following mandibular third molars extraction.

## Introduction

Alveolar Osteitis (AO) is a very important clinical complication that notably occurs following the extraction of mandibular third molars^1^,^2^. It is primarily initiated due to fibrinolysis and disturbance of the process of clot formation.

It has been reported that 20% to 30% is the average incidence after third molar extraction.^3^ Several associated risk factors have been linked here, which may include infection, surgical trauma, the experience of the surgeon, smoking, oral contraceptive use, inadequate blood supply, and poor oral hygiene^4^. Clinical audits and reports have indicated that Antibiotics and antibacterial rinses have been applied as infection is the etiologic factor in the genesis of AO^5^,^6^.

Studies have indicated that Chlorhexidine is an effective antimicrobial rinse for both Gram (+) and Gram (-) organisms. It reduces the quantity of oral microbial organisms and thus may prevent AO^7^, ^8^. On the other hand, studies have shown that beta-lactamase-producing Bacteroides strains were sensitive to a combination of amoxycillin with clavulanic acid (Augmentin)^9^,^10^.

The present study was carried out to ascertain whether Chlorhexidine CHX plus Augmentin can prevent alveolar osteitis after mandibular third molars removal. This study was designed to test the null hypothesis that CHX and Augmentin are not effective in the prevention of AO.

## Materials and methods

This article was written according to the CONSORT guidelines for reporting randomized controlled clinical trials (http://www.consort-statement.org/).

### Study design

This was a three-arm, placebo-controlled, randomized clinical trial. The study was reviewed and approved by the Standing Committee of Bioethics Research, Prince Sattam Bin Abdulaziz University, Alkharj, KSA, approval no. SCBR-078-2022.

### Estimation of the sample size

The sample size was calculated using G* Power 3.1 (Heinrich-Heine-Universität, Düsseldorf, Germany).

A sample of 60 patients in each group would have a 90% power to detect a difference in the means of OA risk factors using the values in a previous trial^6^.

### Eligibility Criteria and Surgery Procedure

Patients fulfilling the following criteria were eligible for inclusion into the study:

- Healthy males and females of age 18 and older
- Patients who have at least one mandibular third molar to be extracted.
- Patients who were willing to participate and co-operate throughout the study and to attend the follow-up appointments and willing to take the full course of treatment. For patients' protection, and to obtain a more uniform sample, the following characteristics were excluded:
- Pericoronitis
- Women who were pregnant, breast-feeding, or using oral contraceptives.
- Hypersensitivity to, or intolerance of, Augmentin or any other Antibiotics.
- Diabetes mellitus
- Current receipt of antibiotics, NSAIDs, systematic corticosteroids or anticoagulants.
- Cardiovascular disease including a history of rheumatic fever, or other conditions requiring antibiotic prophylaxis.

Patients who fulfilled the eligibility criteria were provided with the study information sheets and a patient's written consent was obtained. Only one tooth was removed in one session for this study. Patients first rinsed with 15 mL of 0.2% CHX Gluconate (Avohex, Avalon Pharma) for 15 seconds just before tooth removal. The type of extraction was determined based on the characterization of the tooth. All extractions were performed under local anesthesia with 2% Lidocaine HCl with 1:100000 Epinephrine. Standard surgical procedure was used with copious irrigation of sterile saline. The soft tissues were closed with 3/0 absorbable suture for trans-alveolar procedures.

### Randomization

Patients recruited in the study were randomly allocated into three groups:

#### Group (1)

The day after surgery, the patients began home use of the CHX solution (15 mL for 30 seconds) twice daily for 7 days.

#### Group (2)

In addition to CHX solution, the patients were prescribed Augmentin (500 mg amoxicillin + 125 mg clavulanic acid (GSK Group)); twice daily for 5 days postoperatively.

#### Group (3)

Patients in this control group were provided with sterile saline solution (0.09 % NaCl) as a replacement for CHX. A computer-generated randomization schedule was created, and the randomization codes were enclosed in sealed, opaque and sequentially numbered envelopes. The patient's allocation to either group was revealed by an independent non-participating dental intern following the surgery. The intern was responsible for this randomization including labelling the solutions and tablets bottles. The study was therefore double-blind with neither the surgeons nor the patients being aware of which group the patient was going to be allocated to. In addition, all patients were prescribed a rescue medication of 500 mg Paracetamol (Panadol, GSK Group) for post-operative pain relief. A postoperative examination was performed on the third and seventh day to determine any adverse reactions and assess the presence of AO.

All extractions were performed by two experienced oral surgeons (S. S and MY. K). The time consumed for each surgical procedure and the number of sutures required were recorded and analyzed in relation to a possible incidence of trauma-related OA.

Follow up appointments were arranged at three- and seven-days post extraction. If the patient reported pain unrelieved by analgesics and if exposed bone or necrotic debris were present, the diagnosis of AO was confirmed, and a standard treatment of AO was performed.

### Statistical analysis

SPSS® program Version 13.0 (SPSS Inc., Chicago, USA) was utilized for statistical analysis. The X2 test was used for qualitative variables. To compare the three groups, One-way analysis of variance (ANOVA) or its alternative nonparametric test (i.e., Kruskal-Wallis test) was applied. The level of significance was set at 0.05.

## Results

**Figure 1:**
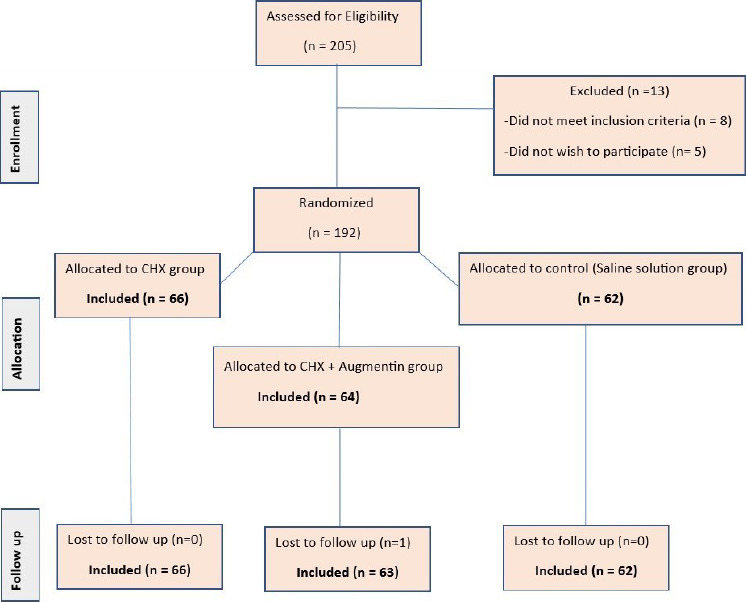
Patients' recruitment and randomization

A total of 191 patients were enrolled and randomized into the assigned three groups (Figure 1). Table 1 and 2 show that the allocated groups were balanced (P > 0.05) in terms of, age, smoking, and the classification of the operated teeth.

**Table 1:** Patients' Groups Description

	Group1	Group2	Group3	p value
**Sex**				0.322
Male	32	31	29	
Female	34	32	33	
**Mean** **age** (year)	25.2	26.1	24.9	0.466
**Smoking**				0.355
Yes	18	13	20	
No	48	50	42	

**Table 2:** Classification of operated teeth and the related Alveolar Osteitis (AO)

Difficulty*	Group 1			Group 2			Group 3		
	AO	Symptomless	Cases	AO	Symptomless	Cases	AO	Symptomless	Cases
Erupted	1	10	11	1	11	12	3	5	8
Impacted *Soft Tissue*	3	13	16	2	11	13	5	15	20
Impacted *Partial Bone*	6	12	18	4	14	18	5	16	21
Impacted Full Bone	3	18	21	1	19	20	2	11	13
**Total**	13	53	66	8**	55	63	15	47	62

* p = 0.288

** p = 0.001

Group 1: CHX (n = 66), Group 2: CHX and Augmentin (n = 63), Group3: Placebo (n = 62).

Erupted teeth in addition to soft tissue, partial bone and full bone impactions represented 16.2%, 25.7%, 29.8% and 28.3% of the extracted teeth respectively [Table 2]. As the surgical trauma has been linked with AO, the number of sutures and the duration of surgery were measured, and the results showed no statistical difference among groups (P > 0.05) [Table 3]. The overall rate of AO was evident in 18.8% of cases, of which 6.8% occurred in group 1; 4.2% in group 2 and 7.8% in group 3. The reduction in AO incidence was statistically significant in group 2 (P<0.05). On the other hand, no statistical significance was found between the classification of the operated teeth-degree of difficulty and the incidence of AO (P > 0.05) [Table 2, 3].

**Table 3:** Further Factors Considered regarding the occurrence of Alveolar Osteitis (AO)

	Group 1	Group 2	Group 3	P value
Total sutures (mean)	4.4	4.3	4.1	0.792
Surgical time (min)	11.3	11	10.7	0.544
Occurrence of AO	13	8	15	0.624

Patients have reported CHD side effects like taste alteration, bad taste, and staining. However, of all the patients in group 1 and group 2, 66.6% and 84.1% respectively were satisfied with the CHD as a mouthwash [Table 4].

**Table 4:** Chlorhexidine CHD unfavourable side effects

Reported Effects	Group 1	Group 2
Taste Alterations	10 (15.2%)	5 (7.9%)
Bad tastes	8 (12.1%)	3 (4.8%)
Staining	4 (6.1%)	2 (3.2%)

## Discussion

Several studies were conducted to limit the occurrence of AO following teeth extraction^11^-^13^. Efforts were concentrated on reducing microorganisms during the process of wound healing and eradicating their significant role in the disruption of the clot formation either through prescribing Antibiotics^14^,^15^, Antiseptic mouthwashes^3^,^16^ or combination of both^6^,^17^,^18^.

Oral infection studies have noted resistant strains of pathogenic microorganisms^19^,^20^ and beta-lactamase-producing bacteroides strains were sensitive to a combination of amoxycillin with clavulanic acid (Augmentin)^21^. Furthermore, Gazal et al., concluded in their randomized controlled trial that the incidence of OA was reduced after surgical extractions when providing preoperative co-amoxiclav with postoperative amoxicillin or metronidazole^22^.

Studies have shown controversy about the effect of CHX on the incidence of AO. Ragno and Szkutnik have reported a statistically significant decrease in the incidence of dry socket in patients using the chlorhexidine rinse with no significant adverse reactions^3^. Larsen has also reported that chlorhexidine was associated with at least a 50% reduction in alveolar osteitis compared with control groups.^23^ Furthermore, studies concluded that the topical application of intra-alveolar bioadhesive chlorhexidine gel may decrease the incidence of alveolar osteitis after removal of third molars^24^,^25^. On the contrary, Krekmanov and Nordenram found that there was non significance difference in the decreased occurrence of AO between the group of CHX alone and the group of combined penicillin and CHX. However, the occurrence of AO seen with both regimens was significantly less than that seen in the control group^17^. Also, the trial of Arteagoitia et al., reported the outcome of pre- and postoperative prophylaxis with no difference between the Antibiotics and the Placebo groups.^18^ Moreover, Monaco et al., found that postoperative amoxicillin administration did not have a significant effect on AO prevention after third molar surgery^26^.

In the present study, there was a statistically non-significant decrease in the occurrence of AO in group 1 (CHX). However, group 2 (CHX + Augmentin) had a significantly lower occurrence of AO. These results correspond with the study of Delilbasi et al.^6^. However, the later trial was not double-blind research. The current findings suggest that CHX alone might be ineffective in reducing the occurrence of AO and the bacterial levels after rinsing may still be high enough to initiate bacterial fibrinolysis^27^. A very recent study on the effect of ozone gas and 1% CHX gel on the incidence of dry socket has shown that both have reduced the symptoms of AO and ozone gas acquired better prevention means^28^.

CHX adverse reactions like oral tissues staining always form the main concerns for the patients^29^,^30^. Most of the adverse reactions involved in the current study were bad taste; alterations in sense of taste during treatment and tissue staining. While the latter can be overcome by oral cleansing, taste alteration issue was solved after completing the specified period of CHX use.

In Conclusion, the combination of CHX plus Augmentin may be useful in reducing the incidence of AO following mandibular third molars extraction. Additional studies are required to assess the effects of various antimicrobial rinses and antibiotics alone or in combination on the prevention of Alveolar Osteitis.

## Abbreviations

CHX: Chlorhexidine
AO: Alveolar Osteitis
